# Fournier's Gangrene During Lenvatinib Treatment for Hepatocarcinoma

**DOI:** 10.7759/cureus.82881

**Published:** 2025-04-24

**Authors:** Simone Rota, Marco de Scordilli, Riccardo Vida, Michela Guardascione, Paola Di Nardo, Arianna Fumagalli, Adrian Zdjelar, Stefania Bottos, Paolo Cabas, Federica Maffeis, Elena Ongaro, Luisa Foltran, Fabio Puglisi

**Affiliations:** 1 Department of Medical Oncology, Centro di Riferimento Oncologico di Aviano (CRO) IRCCS, Aviano, ITA; 2 Department of Medicine, University of Udine, Udine, ITA; 3 Department of Oncologic Radiation Therapy and Diagnostic Imaging, Centro di Riferimento Oncologico di Aviano (CRO) IRCCS, Aviano, ITA; 4 Department of Hospital Medical Management, Santa Maria degli Angeli Hospital (ASFO), Pordenone, ITA; 5 Department of Complex Structure of Urology, Santa Maria degli Angeli Hospital (ASFO), Pordenone, ITA; 6 Department of General Surgery, Santa Maria degli Angeli Hospital (ASFO), Pordenone, ITA

**Keywords:** adverse event, antiangiogenic drugs, fournier's gangrene, gastrointestinal oncology, hepatocarcinoma, lenvatinib, necrotizing fasciitis, toxicity, tyrosine kinase inhibitors

## Abstract

Antiangiogenic drugs such as lenvatinib have demonstrated significant benefits in patients with hepatocarcinoma (HCC), with an acceptable safety profile. However, serious side effects have been documented, though rare. In this report, we describe the case of a severe and unexpected toxicity encountered after about seven months of lenvatinib treatment. The patient developed a septic state, widespread cutaneous erythema with extensive necrotic involvement of the pelvic floor, with a diagnosis of Fournier's gangrene (FG). Emergency surgery, including sigmoidostomy and wide necrosectomy, was necessary, and further surgeries were performed in the following days due to persistent necrotic tissue. FG is a rare form of necrotizing fasciitis that has been described as rarely associated with several antiangiogenics, even in the absence of major risk factors. Cases of FG have been rarely documented in association with lenvatinib treatment, and this is the first report on a European HCC patient receiving an 8 mg daily dose. Considering the ever-growing use of antiangiogenics in HCC patients and their clinical complexity, it is crucial to be vigilant even about rare toxicities like FG, especially with known concomitant risk factors. Careful monitoring and a multidisciplinary approach are fundamental to promptly identify and address potentially life-threatening complications.

## Introduction

Lenvatinib is a multireceptor tyrosine kinase inhibitor (TKI), a class of drugs that block the activity of enzymes involved in cell signaling and growth, and is approved for the treatment of several solid tumors, including radioiodine-refractory thyroid cancer, renal cell carcinoma, and endometrial cancer. It is also approved for the first-line treatment of advanced/inoperable hepatocarcinoma (HCC), defined as Barcelona Clinic Liver Cancer (BCLC) stage C, as well as for localized HCC progressed to locoregional strategies (BCLC stage B) [1]. In the advanced setting, lenvatinib represents the main alternative to therapeutic combination regimens involving immune checkpoint inhibitors. These approvals are for patients with a preserved liver function (class A in the Child-Pugh Score, used to clinically evaluate the prognosis of patients with chronic liver disease based on bilirubin and albumin blood concentration, international normalized ratio (INR), and the presence of ascites and hepatic encephalopathy) and a good performance status (PS 0-1 in the ECOG scale developed by the Eastern Cooperative Oncology Group to assess patients' level of functioning in their daily activities). 

Lenvatinib targets multiple tyrosine kinase receptors involved in pathological angiogenesis, tumor growth, and cancer progression. These include the vascular endothelial growth factor (VEGF) receptors 1-3, the fibroblast growth factor receptors (FGFR) 1-4, the platelet-derived growth factor receptor (PDGFR) alpha, KIT, and RET [2]. Notably, antiangiogenic drugs such as TKIs and monoclonal antibodies like bevacizumab have demonstrated significant benefits in HCC patients, maintaining an acceptable safety profile. Specifically, the most commonly reported adverse events with lenvatinib in registration trials for HCC (at a dose of 12 mg daily or 8 mg daily for patients weighing less than 60 kg) include hypertension (42%), diarrhea (39%), hyporexia (34%), weight decrease (31%), fatigue (30%), palmar-plantar erythrodysesthesia (27%), proteinuria (25%), dysphonia (24%), and nausea (20%) [1,3]. However, several serious side effects have also been documented, such as liver failure, hepatic encephalopathy, and hemorrhages.

In this report, we describe a case of a severe and unexpected toxicity encountered during lenvatinib treatment in an HCC patient at our institute, with the aim of raising awareness on rare adverse events and helping their clinical monitoring, to optimize patients' outcomes.

## Case presentation

A 68-year-old man was referred to the National Cancer Institute in Aviano following a new diagnosis of multifocal bilobar well-differentiated HCC, not suitable for locoregional strategies (BCLC stage C). The patient had preserved hepatic function and good performance status (ECOG PS 1); HCC was attributed to multifactorial etiology: exotoxic, due to alcohol abuse, and dysmetabolic, due to metabolic syndrome and type 2 diabetes mellitus. In December 2022, he commenced treatment with lenvatinib at a reduced dose of 8 mg daily, considering his age, PS, and comorbidities. Initially, he reported no adverse events except for grade 1 asthenia and dysphonia. Given his good tolerance, the dose was increased to 12 mg daily. However, after two cycles, he experienced a worsening of asthenia and developed grade 2 hand-foot syndrome and hypothyroidism necessitating replacement therapy. The dose was subsequently reduced back to 8 mg, with no further unexpected toxicities. In March and June 2023, CT scans showed a partial response to the treatment, concomitant with a slight decrease in serum alpha-fetoprotein levels.

**Figure 1:**
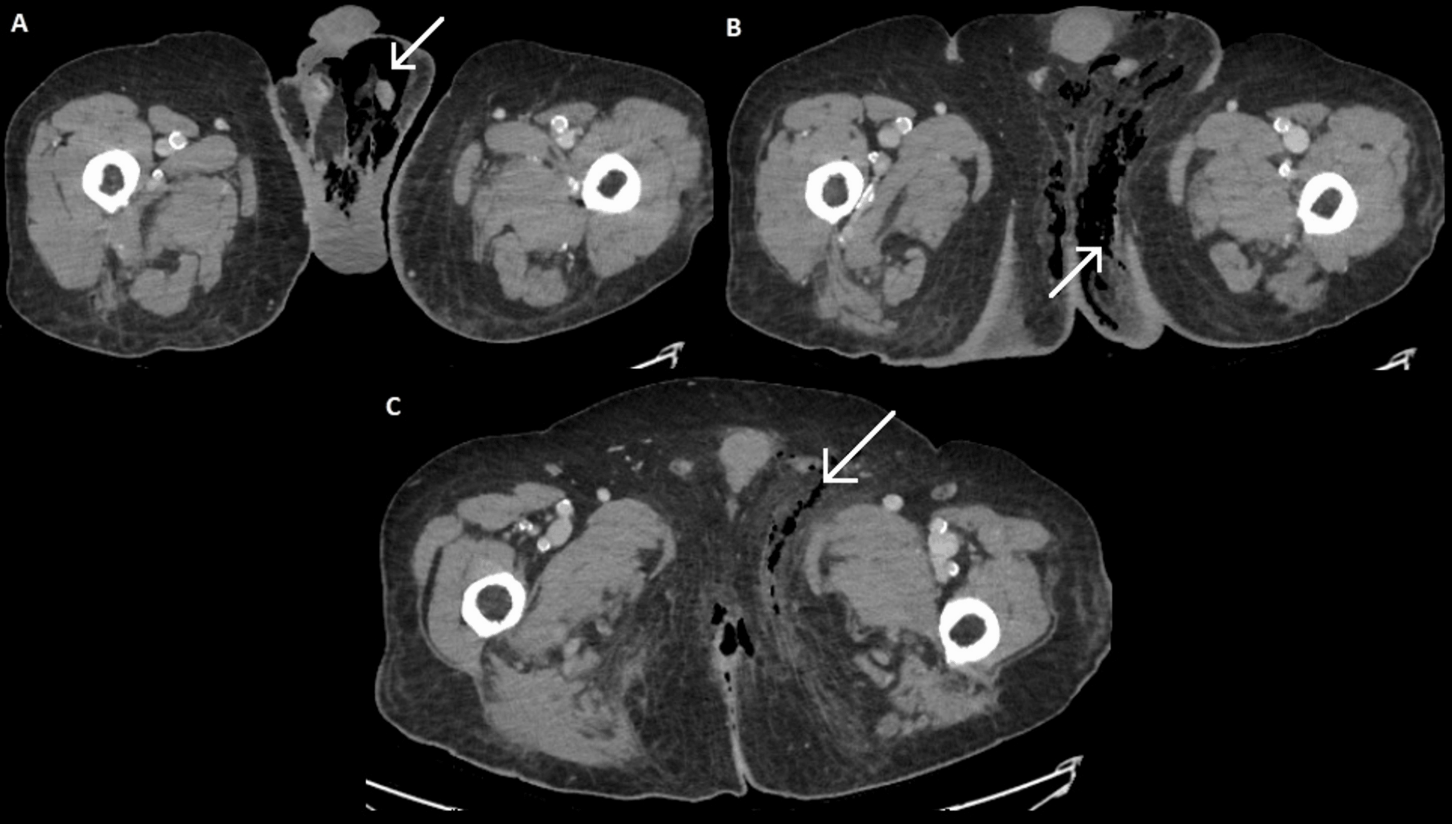
CT scans revealing extensive necrotic involvement of the pelvic floor. CT scan images in portal venous phase showing extensive gas within the scrotum (A) and ischioanal fossae (B), extending superiorly into the left inguinal canal (C).

In July 2023, however, the patient developed a fever unresponsive to antipyretics and grade 2 diarrhea, leading to emergency service after about 10 days of persistent symptoms. He exhibited widespread cutaneous erythema, particularly in the perineal region, which he had noticed only for a couple of days. Necrosis was visible on the left gluteus, pneumoscrotum and a perianal fistula were observed, and he was hospitalized on July 19. Considering the extension, depth, and localization of the necrotic areas, as well as the rapidly evolving clinical situation, a diagnosis of left perineal and scrotal Fournier's gangrene (FG) was made, grade 4, according to the Common Terminology Criteria for Adverse Events (CTCAE, Version 5.0) and most likely related to the angiogenic effect of lenvatinib. Apart from well-controlled diabetes mellitus (managed with metformin and gliclazide, with a glycated hemoglobin lower than 7%), no other major risk factors for FG were identified, though previous alcohol abuse and mild obesity were noted as potential risk factors. An abdominal CT scan revealed extensive necrotic involvement of the pelvic floor (Figure 1).

**Figure 2:**
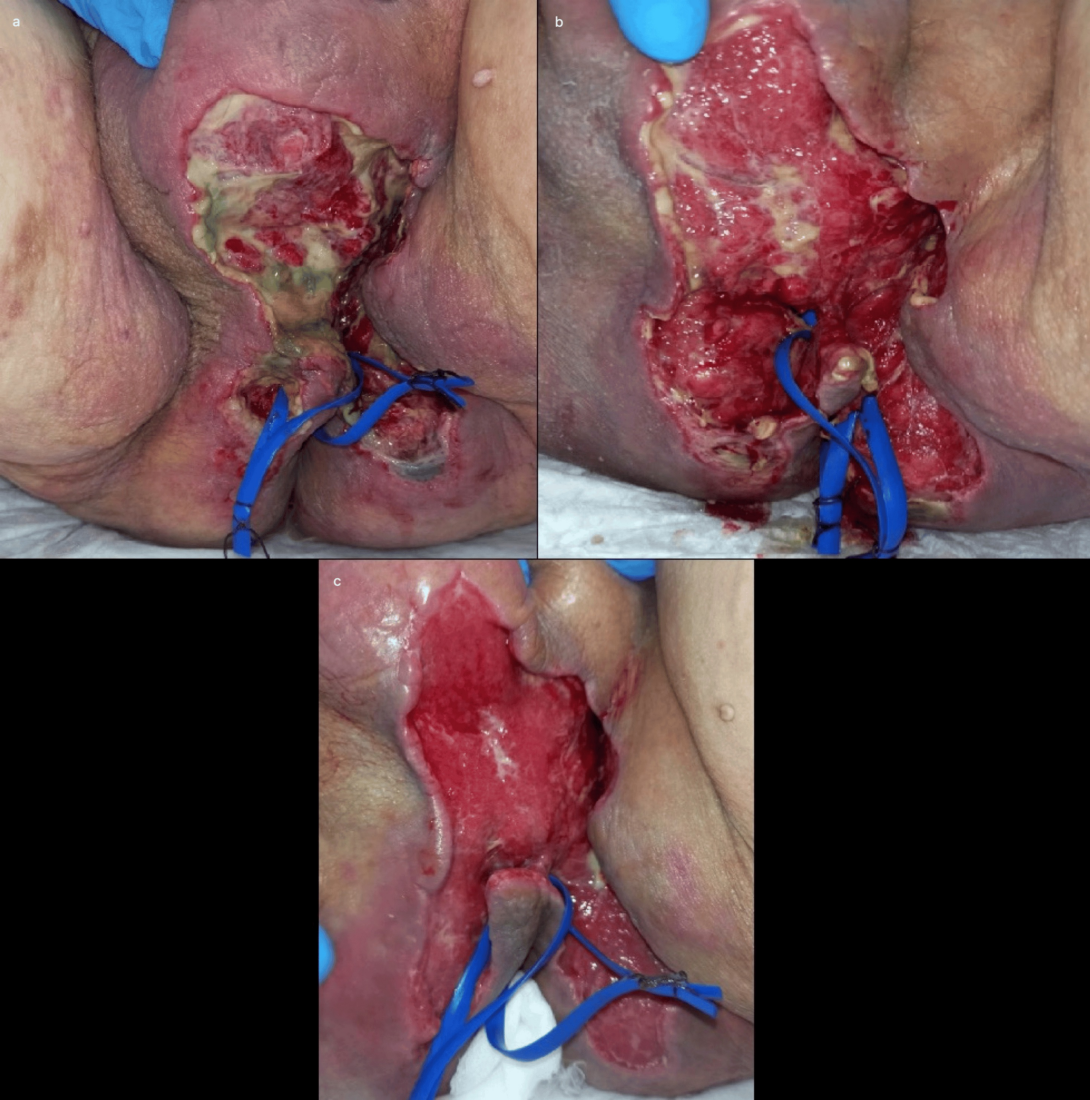
Gradual improvement of the perineal situation after multiple surgeries. Gradual local improvement after the last surgical debridement (July 24, 2023) due to the persistence of necrotic tissue in the scrotal region. Perineal situation on July 28 (a), August 7 (b), and August 17 (c).

Microbiological analysis from the purulent specimens identified two microbial cultures positive for *E. coli* and *E. faecalis* (sensible to almost all tested antibiotics). A multidisciplinary collaboration was fundamental for a proper and rapid management of this relatively rare condition. Following prompt urologic and surgical examinations, the patient underwent immediate emergency surgery (about 10 days after the first clinical symptoms) including a lateral videolaparoscopic sigmoidostomy, wide necrosectomy, and perineal toilet. A suprapubic cystostomy was not deemed necessary considering the progression of the necrotic extension towards the posterior region of the pelvis. Further surgeries on July 22 and 24 addressed persistent necrotic tissue in the scrotal region. At the same time, an infectious disease specialist consultation was needed. Initially, intravenous ceftriaxone and metronidazole were administered; due to a worsening of the necrotic area, from July 24, antibiotic therapy was escalated to piperacillin/tazobactam (4.5 g as a loading dose and then 18 g as continuous daily infusion) and tigecycline (100 g as a loading dose and then 50 g every 12 hours). Continued intravenous antibiotic treatments were needed for the complete resolution of the infectious complications, leading to a slow, gradual improvement of the perineal situation (Figure 2).

Tigecycline was suspended from August 7, while piperacillin/tazobactam was continued until August 14. No reconstructive interventions were needed, thanks to the good local healing. The clinical course was further complicated by liver failure, prompting several hepatological assessments. 

Despite these challenges, the patient's clinical condition gradually improved, and he was discharged on August 17, 2023, with a permanent colostomy after nearly a month of hospitalization. Lenvatinib treatment was discontinued following the FG diagnosis, and he was maintained on best supportive care. In May 2024, due to progressive deterioration of his clinical conditions, he was admitted to a local hospice, where he died on June 14. His death was in all likelihood related to progressive HCC; however, the serious adverse event changed his clinical course and contraindicated further treatment lines, both due to his worsened performance status and due to the impossibility of administering other TKIs with antiangiogenic effect.

## Discussion

Lenvatinib has seen increasing use across several solid tumors and continues to play a pivotal role as a treatment of choice in HCC patients not eligible for first-line treatment with immune checkpoint inhibitors, particularly those with non-viral etiology [4]. Moreover, the use of antiangiogenic drugs in HCC patients is expected to grow, also in combination with locoregional treatment strategies such as transcatheter arterial chemoembolization (TACE), given the clinical benefits demonstrated by recent clinical trials [5-7]. 

FG is a form of necrotizing fasciitis, a severe soft tissue infection that primarily affects the male external genitalia and the pelvic floor. As a urologic emergency, FG necessitates the immediate initiation of intravenous antibiotic therapy, hemodynamic support with fluid infusion, rapid surgical debridement to remove necrotic tissue, as well as colostomy and possibly suprapubic cystostomy diversions. Despite prompt antibiotic and surgical interventions, the mortality rate remains alarmingly high, ranging from 18% to 30% [8,9] and up to 40% in some retrospective datasets [10,11].

FG is not exceedingly rare and often arises as a complication of uncontrolled diabetes mellitus in elderly male patients, particularly those with concurrent alcohol abuse or in individuals who are malnourished or immunocompromised [12]. Additional risk factors include local trauma, surgical procedures, and obesity. Necrotizing fasciitis, including FG, is recognized as a rare adverse event associated with antiangiogenic drugs such as bevacizumab, ramucirumab, aflibercept, and regorafenib, with several cases reported in the literature [13-22]. To date, only two other cases of FG have been documented in association with lenvatinib treatment [23,24]. Patients' characteristics are summarized in Table 1. It is important to note that FG can develop even in the absence of major risk factors.

**Table 1:** Patients' characteristics from case reports of necrotizing fasciitis and FG with antiangiogenic drugs. FG: Fournier's gangrene; M: male; L: line; NR: not reported; HCC: hepatocarcinoma; RT: radiation therapy; F: female

Reference	Age	Sex	Tumor	Setting	Risk factors	Prior surgery	Antiangiogenic drug	Onset time
Barone et al. [23]	80	M	Thyroid	Metastatic (1L)	No	No	Lenvatinib	14 months
Fukuhisa et al. (abstract only) [19]	73	M	Rectum	Metastatic (1L)	NR	No	Bevacizumab	NR
Gamboa et al. [17]	67	M	Colon	Adjuvant	No	Yes (hemicolectomy)	Bevacizumab	10 months
Gonzaga-López et al. [16]	64	M	Sigma/rectum	Metastatic (>3L)	Yes (hypertension, ex-smoker, mild drinker)	Yes (sigmoidectomy)	Aflibercept	23 days
Komeda et al. [24]	66	M	HCC	Metastatic (1L and 2L)	Yes (diabetes, obesity, paralysis of the lower half of the body)	No	Bevacizumab and then lenvatinib	4 months of bevacizumab, 4 weeks of lenvatinib
Koyama et al. (abstract only) [15]	66	M	Rectum	Metastatic (2L)	NR	Yes (sigmoid colostomy)	Bevacizumab	14 days
Koyama et al. (abstract only) [15]	63	M	Rectum	Metastatic (5L)	NR	Yes (sigmoid colostomy)	Regorafenib	16 days
Imamura et al. (abstract only) [13]	52	M	Rectum	Metastatic (2L)	NR	Yes (abdominoperineal resection)	Bevacizumab	2 months
Ishida et al. (abstract only) [14]	51	M	Rectum	Metastatic (1L)	Yes (palliative RT (30 Gy))	Yes (abdominoperineal resection)	Bevacizumab	3 months
Rakusic et al. [18]	76	M	Stomach	Metastatic (1L)	No	Yes (gastrectomy, omentectomy, splenectomy, subtotal pancreatic resection, esophagojejunostomy, entero-enteral anastomosis Roux)	Ramucirumab	1 month
Sendur et al. [20]	49	M	Rectum	Metastatic (1L)	NR	Yes (low anterior resection with ileostomy)	Bevacizumab	40 days
Ugai et al. [21]	59	F	Rectum	Metastatic (1L)	Yes (palliative RT (30 Gy))	Yes (low anterior resection)	Bevacizumab	6 months

The pathophysiology of FG is complex, influenced by several factors that contribute to its development. Three key elements play a crucial role in the onset and rapid progression of this life-threatening condition, alongside host predispositions. FG is typically a polymicrobial infection involving both aerobic and anaerobic microorganisms (type I), necessitating the use of broad-spectrum antibiotics; monomicrobial FG is rare (type II) [25]. Also, identifying an entry point for the infective source in a usually confined anatomical space is crucial for understanding FG's development. Common sources of infection are the anorectum, urogenital tract, and skin of the genitalia [26]. Complications such as anal fissures, fistulas, and colonic perforations have also been described. Furthermore, the impairment of healing mechanisms is primarily responsible for the rapid progression of the destructive processes associated with FG, often forming a vicious cycle.

Antiangiogenic drugs might impact each of these factors. For instance, an increased risk of infections has been associated with bevacizumab, and an even higher incidence with other agents such as aflibercept [27]. Regarding the second point, anti-VEGF/VEGFR drugs significantly increase the risk of bowel perforation, with reported incidence rates up to 5% [28,29]. Drugs such as regorafenib and ramucirumab have also been linked to the development of bowel perforation [30,31]. Various mechanisms have been proposed for delayed healing and the inhibition of angiogenesis. Anti-VEGF agents cause the direct regression of normal blood vessels and reduce nitric oxide release, leading to a decreased oxygen supply; additionally, endothelial damage triggers the coagulation cascade, contributing to disseminated microthromboses, ischemia, and necrosis of the fascia [8]. Lenvatinib, as a multiple kinase inhibitor, is associated with ischemic changes and decreased microvessel density, inflammatory cell infiltration, and intratumoral hemorrhage and necrosis, both in vitro and in vivo [32-34]. 

Our patient developed complications approximately seven months after starting lenvatinib, with a rapidly evolving clinical course. Microbiological analysis identified two microbial cultures from the purulent specimens, positive for *E. coli* and *E. faecalis*. A CT scan pinpointed a perianal fistula, identifying it as the most likely infective source of infection. 

Data on lenvatinib and gastrointestinal perforation/fistulas have been documented in regulatory trials. Fistulas in the respiratory and digestive tracts are not uncommon in thyroid cancer patients. However, it must be noted that in these cases, lenvatinib doses are typically higher (24 mg per day) and most complications have been reported with concomitant risk factors, such as previous or concurrent radiotherapy. A case of bowel perforation in a patient treated with lenvatinib for HCC has been reported [35], and specific anatomopathological features on the surgical specimen raised suspicions of drug-related effects. Unfortunately, due to the rapid clinical progression in our case, such features were not identifiable. As for the healing process, our patient's diabetes, a condition known for altered vascularization and an immunosuppressive state, likely contributed to the delayed recovery of injuries, exacerbating the pathophysiology of FG despite adequate glycemic control. The patient's diabetes probably contributed also to the slow resolution of the infection, even if glycemic levels were adequately controlled during the hospitalization, despite the septic state. 

Considering the severity and the rapid evolution of FG, prompt recognition is fundamental to ensure the adequate management of this deadly clinical condition. The administration of lenvatinib, and of antiangiogenics in general, requires particular caution especially in the presence of risk factors such as diabetes, immune deficiencies, or other conditions predisposing to polymicrobial infections (i.e., recent surgery). The onset of suspect erythema or signs of cutaneous infection must be considered as clinical red flags, in particular with concomitant risk factors; potential sources of infection, such as perianal fistulas, should be excluded. The recognition of the first signs of infection should imply a prompt drug discontinuation. Simple dose adjustments are difficult to consider, bearing in mind the severity and rapid evolution of this kind of adverse events, and definitive discontinuation is advisable. Also, evaluating other treatment lines can be challenging, considering the persistence of risk factors and the typical worsening of patients' performance status. Other TKIs can't be administered in view of their antiangiogenic effect, and patients surviving serious adverse events such as FG are maintained on best supportive care.

## Conclusions

This case represents one of the rare reports of FG likely associated with lenvatinib treatment and is the first documented case in a European patient with HCC treated with an 8 mg daily dose. Considering the increasing use of antiangiogenic drugs and the clinical complexity of HCC patients, it is crucial to be vigilant even about rare toxicities like FG, especially with known concomitant risk factors. This case highlights the importance of careful monitoring and a multidisciplinary approach in managing lenvatinib therapies to promptly identify and address potentially life-threatening complications that can also lead to permanent treatment discontinuation. Particular attention must be given to the emergence of fever unresponsive to antipyretics, gastrointestinal or urological symptoms, or widespread cutaneous erythema. The rapid involvement of each specialist is fundamental, considering that an aggressive management of FG, combining broad-spectrum antibiotics and surgical debridement, remains essential for improving clinical outcomes. Further studies are needed to explore the mechanisms underlying these rare complications and to optimize the safety of antiangiogenic therapies in HCC patients.
